# Western Medical Society of Wisconsin

**Published:** 1850-09

**Authors:** Geo. D. Wilber

**Affiliations:** Rec. Sec’y.


### ARTICLE XII.

### THE WESTERN MEDICAL SOCIETY

Of the State of Wisconsin met in this place on Tuesday, the 4th inst., and although the weather was bad, there was a full attendance.

The President being absent, Dr. H. Van Dusen was called to the Chair. Several new members were admitted, and others made application for Diplomas, but only one was granted.

The resignations of Dr. G. W. Phillips, Treasurer, and Dr. W. Clark, Censor, were received. Dr. G. W. Bunnall, of Dodgeville, was elected Treasurer, in place of Dr. Phillips, resigned. Dr. D. Ross was elected Censor, in place of Dr. Clark, resigned.

The following Standing Committees were created, and the President made the following appointments to the same :—

*On Topographical Medicine.*---Dr. A. Sampson, Chairman, Dr. D. Ross, and Dr. J. S. Russell.

*On the Condition of the Medical Profession in this District.*—Dr. Geo. D. Wilber, Chairman, Dr. C. A. Mills, and Dr. Wm. Stoddard.

*On Indigenous Botany*---Dr. G. W. Bunall, Chairman, Dr. R. F. Hays, and Dr. T. R. Kibbe.

*On Essays.*---Dr. John Cassels, Chairman, Dr. A. P. Ladd, and Dr. J. I. Bayse.

The following resolution was unanimously adopted :—

*Resolved*, That no person shall be eligible to the studentship of medicine, who has not a good English education; such an acquaintance with the Greek and Latin languages as will enable him to appreciate the technology of medicine, and who does not maintain a reputable moral character, and possess good mental endowments.

Article XIV. of the By-Laws reads in this wise :—

"It shall be the duty of the Corresponding Secretary to notify all persons engaged in the practice of medicine or surgery within the limits of this Society, not already members thereof, as soon may be expedient, of the existence and objects of our organization, and invite each to become a member, and if, after being thus notified for six months, they shall neglect, refuse, or fail to become members, they shall be deemed irregular practitioners; and it shall be improper for any member of this Society, in his professional capacity, to advise or consult with such irregular practitioner."

On motion, the Recording Secretary was instructed to have an abstract of the proceedings of this meeting published in the Wisconsin Tribune.

Dr. Van Dusen was elected one of the delegates to attend the meeting of the Wisconsin State Medical Society.

On motion, the Society adjourned to meet in Hazel Green on the first Tuesday of December, 1850.

GEO. D. WILBER,
*Rec. Sec'y.*

